# Subjective Dry Eye Symptoms and Objective Ocular Surface Signs in a Civil Air Crew Population: A Cross-Sectional Study

**DOI:** 10.7759/cureus.51447

**Published:** 2024-01-01

**Authors:** Anna Fachinetti, Edoardo Marelli, Paola Velati, Piercarlo Minoretti, Giuseppe De Palma, Camilla Sigurtà

**Affiliations:** 1 Aviation Medicine, Cavok Medical Center, Lonate Pozzolo, ITA; 2 Medical and Surgical Specialties, Radiological Sciences, and Public Health, University of Brescia, Brescia, ITA; 3 Occupational Health, Studio Minoretti, Oggiono, ITA

**Keywords:** schirmer’s-1 test, tear film break-up time, speed questionnaire, dry eye disease, aircrew personnel

## Abstract

Background

Aviation professionals are often exposed to conditions such as low cabin air pressure, reduced humidity, and prolonged artificial lighting, which may predispose them to dry eye disease (DED). We therefore designed a cross-sectional study with three primary objectives. Our first aim was to determine the prevalence of subjective dry eye symptoms among civil flight personnel. To achieve this, we administered the Standard Patient Evaluation of Eye Dryness (SPEED) questionnaire. Second, we performed ocular examinations to assess objective DED indicators, such as the Schirmer’s-1 (SCH-1) test and tear film break-up time (TBUT). We then correlated the results of these objective tests with the subjective symptoms reported by the participants. Last, we aimed to identify the independent risk factors for positive SCH-1 and TBUT results among aircrew personnel who did not report subjective dry eye symptoms.

Methods

The study sample consisted of 189 aircrew personnel (94 men and 95 women; mean age: 35.8 ± 10.4 years). They completed the SPEED questionnaire, a tool for assessing ocular surface symptoms. Participants' symptoms were classified as normal (SPEED scores 0-6), moderate (SPEED scores 7-15), or severe (SPEED scores 16-28). The objective assessment included the SCH-1 test for tear production and the TBUT test for tear film quality.

Results

A significant majority of participants (n = 165; 87.3%) did not report any subjective symptoms of DED. However, 12.2% (n = 23) and 0.5% (n = 1) of the study subjects experienced moderate and severe symptoms, respectively. The SCH-1 test and TBUT test were positive in 25.4% (n = 48) and 24.9% (n = 47) of the participants, respectively. Interestingly, among the aircrew personnel who did not report any subjective dry eye symptoms (SPEED scores 0-6), 18.8% (n = 31) and 17.6% (n = 29) showed abnormal results on the SCH-1 test and TBUT, respectively. Age was identified as the only independent predictor of a positive TBUT (odds ratio = 1.05, 95% confidence interval (CI) = 1.01-1.08, p = 0.01), with a borderline significant association with a positive SCH-1 test (odds ratio = 1.03, 95% CI = 0.99-1.07, p = 0.06).

Conclusions

The disparity between subjective symptoms and objective tests emphasizes the significance of incorporating objective measures for screening and diagnosing DED in civil flight personnel. If independently confirmed by future research, our findings could potentially lead to the routine implementation of surveillance protocols that incorporate objective DED indicators. Moreover, as age emerged as an independent predictor of positive results on objective tests, it is crucial to consider age-specific screening strategies.

## Introduction

Dry eye disease (DED) is a common health issue globally, affecting between 10% and 30% of the general population, and it imposes significant financial costs on individuals and society [1,2]. This multifactorial disorder is characterized by an imbalance in tear film stability, which can lead to inflammation and damage to the ocular surface [3,4]. The Standard Patient Evaluation of Eye Dryness (SPEED) questionnaire has been recognized as an effective method for assessing the subjective symptoms of dry eye, such as their frequency and intensity [5,6]. Nonetheless, the complex nature of DED, often characterized by a discrepancy between subjective symptoms like discomfort, pain, grittiness, redness, dryness, foreign body sensation, and ocular surface signs, complicates its diagnosis and treatment [7,8].

Recent research has underscored the significant role of occupational factors in the onset of DED. For instance, it has been reported that office workers who spend more than seven hours a day using computers often experience a significant reduction in tear film stability and tear production [9], which are key indicators of DED. Similarly, workers in industries such as marble and metal have been found to have a high prevalence of DED [10]. Furthermore, certain occupational groups, including those involved in construction and machinery operations, have been identified as having an increased risk of developing this condition [11]. These findings highlight the importance of considering job-related factors in the prevention, diagnosis, and treatment of DED.

In aviation personnel, factors like cabin air pressure, reduced humidity, and extended exposure to artificial light could potentially lead to the onset or exacerbation of DED [12]. However, aside from a study involving 1246 Australian commercial pilots, where 72.3% reported DED symptoms [13], there is a scarcity of research in this field. Despite the frequent use of artificial tears by flight personnel to counteract the aircraft's low humidity [14], the effects on ocular surface indicators have not been extensively studied. As a result, the relationship between subjective symptoms and objective observations remains ambiguous.

This prompted us to design a cross-sectional study with three main objectives. Our first aim was to determine the prevalence of subjective dry eye symptoms among civil flight personnel. To achieve this, we administered the SPEED questionnaire [5,6]. Second, we performed ocular examinations to assess objective DED indicators, such as the Schirmer’s-1 (SCH-1) test [15] and tear film break-up time (TBUT) [16]. We then correlated the results of these objective tests with the subjective symptoms reported by the participants. Last, we aimed to identify the independent risk factors for positive SCH-1 and TBUT results among aircrew personnel who did not report subjective dry eye symptoms.

## Materials and methods

Ethics

This cross-sectional, observational research adhered to the ethical guidelines outlined in the Declaration of Helsinki and was approved by the Local Ethics Committee of Studio Minoretti (reference number: DE/CMC/2022). Prior to their inclusion in the study, all participants provided their written informed consent.

Study settings and participants

The study was conducted at the Cavok Medical Center, an Italian Civil Aeromedical Center located at Malpensa Airport, Italy, which serves as the primary international airport for the Milan metropolitan area. In 2022, Malpensa Airport boasted a passenger volume of 21.3 million, making it the 23rd busiest airport in Europe. The research took place between September and December 2022, focusing on a convenience sample of civil aircrew personnel who were undergoing routine fitness-for-flight assessments. We excluded individuals who met any of the following criteria: being under 18 years old; having significant ocular or systemic conditions, including eyelid deformities, facial nerve paralysis, or Sjögren's syndrome; having undergone ocular procedures such as corneal refractive surgery in the past six months; using contact lenses; taking any ocular or systemic medications; or using eye drops.

SPEED questionnaire

**Figure 1:**
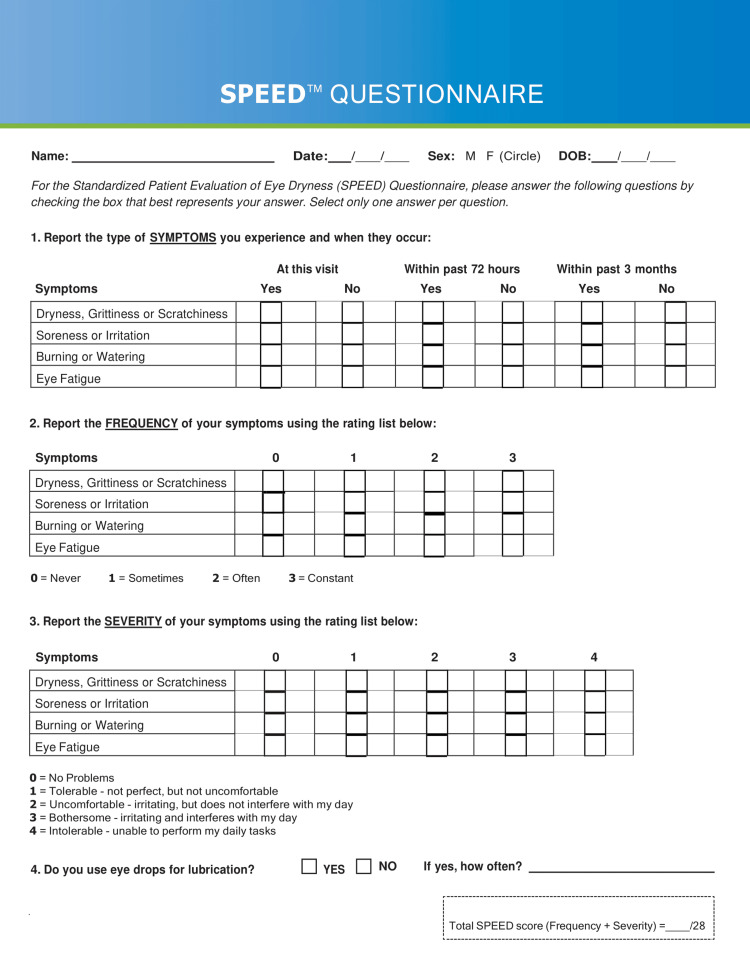
Standard Patient Evaluation of Eye Dryness (SPEED) questionnaire

All subjects filled out the SPEED questionnaire (Appendix: Figure 1), a common method for evaluating symptoms of the ocular surface [5,6]. This survey asks about various symptoms such as dryness, grittiness, irritation, burning, watering, and eye fatigue. Symptoms were rated based on their frequency (0-3) and severity (0-4), with higher totals indicating more severe or frequent symptoms. The sum of these scores yielded a composite score between 0 and 28. Participants were then classified into categories of normal (0-6), moderate (7-15), or severe (16-28) dry eye symptoms according to their composite score. This scoring approach is consistent with established guidelines in the literature [17].

Ophthalmological assessment

An expert ophthalmologist (PV) performed eye examinations using a portable hand biomicroscope (Portable Slit Lamp, Reichert, Inc., New York, United States) under standard lighting conditions. To maintain consistency and minimize the effects of diurnal variations in the tear film layer, these evaluations were conducted between 1:00 PM and 3:00 PM. The examination protocol incorporated a SCH-1 test to gauge tear production [15] and a TBUT test to assess the quality of the tear film [16]. The SCH-1 test procedure involved the placement of a standard Schirmer’s test filter strip in the lower fornix without the use of anesthesia. After a duration of five minutes, the extent of wetting was measured in millimeters. A result of less than 10 mm was deemed abnormal [18]. Before the TBUT measurement, the precorneal tear film was stained with fluorescein. The patient was then examined under a slit lamp using red-free illumination (blue filter). The time interval from the moment the eyelid opened to the appearance of the first dry spots on the cornea was measured using a stopwatch. The TBUT value was calculated as the average of three such measurements. A TBUT value of less than 10 seconds was considered abnormal [19]. To avoid inducing excessive tear reflex secretion, direct contact with the cornea was avoided during the examination.

Data analysis

The study data were summarized using descriptive statistics, including counts, percentages, means, and standard deviations. The chi-squared test was used to compare categorical variables. The Spearman’s correlation coefficient (ρ) was used to analyze the associations between continuous variables. A multivariable logistic regression analysis was conducted to identify independent risk factors for positive results in ophthalmological assessment among subjects with SPEED scores ranging from 0 to 6, indicating no subjective dry eye symptoms. The results are presented as adjusted odds ratios (ORs) along with their 95% confidence intervals (CIs). Analyses were performed using IBM SPSS Statistics for Windows, Version 20 (Released 2011; IBM Corp., Armonk, New York, United States), and all tests were two-sided with a significance level of 5%.

## Results

Subjective dry eye symptoms

A total of 189 aircrew personnel (mean age: 35.8 ± 10.4 years; 94 men and 95 women) were evaluated for subjective dry eye symptoms using the SPEED score. The majority of the participants, 165 (87.3%), with an average age of 35.5 ± 10.5 years (comprising 80 men and 85 women), showed no subjective dry eye symptoms, as their SPEED scores ranged between 0 and 6. In a smaller group, 23 participants (12.2%), with an average age of 37.4 ± 9.4 years (13 men and 10 women), had moderate dry eye symptoms with SPEED scores between 7 and 15. Only one male participant (0.5%), aged 40, had a severe dry eye score of 18. We found no significant correlations between the SPEED score and age (ρ = 0.065, p = 0.38) or sex (ρ = -0.091, p = 0.21).

SCH-1 test

A total of 48 (25.4%) study participants had a positive SCH-1 test, indicating insufficient tear production. Table 1 shows the SCH-1 results in relation to subjective dry eye symptoms (SPEED scores). As expected, the prevalence of a positive SCH-1 test differed significantly according to the subjective severity of dry eye symptoms (p < 0.001). However, 18.8% of aircrew personnel with SPEED scores between 0 and 6 had an abnormal SCH-1 test.

**Table 1:** Schirmer’s-1 test results in relation to subjective dry eye symptoms measured with SPEED scores SCH-1: Schirmer’s-1

	No subjective dry eye symptoms (n = 165; 87.3%)	Moderate dry eye symptoms (n = 23; 12.2%)	Severe dry eye symptoms (n = 1; 0.5%)	p-value
SCH-1 ≥ 10 mm (negative)	134 (81.2%)	7 (30.4%)	0 (0%)	<0.001
SCH-1 < 10 mm (positive)	31 (18.8%)	16 (69.6%)	1 (100%)	

TBUT test

A total of 47 (24.9%) study participants had a positive TBUT test, indicating that the tear film on the surface of the eye was breaking up faster than normal. Table 2 shows the TBUT results in relation to subjective dry eye symptoms (SPEED scores). As expected, the prevalence of a TBUT test < 10 seconds differed significantly according to the subjective severity of dry eye symptoms (p < 0.001). Notably, 17.6% of aircrew personnel with SPEED scores between 0 and 6 had an abnormal TBUT test.

**Table 2:** Tear film break-up time test results in relation to subjective dry eye symptoms TBUT: Tear film break-up time

	No subjective dry eye symptoms (n = 165; 87.3%)	Moderate dry eye symptoms (n = 23; 12.2%)	Severe dry eye symptoms (n = 1; 0.5%)	p-value
TBUT ≥ 10 seconds (negative)	136 (82.4%)	7 (26.1%)	0 (0%)	<0.001
TBUT < 10 seconds (positive)	29 (17.6%)	17 (73.9%)	1 (100%)	

Independent predictors of positive SCH-1 and TBUT results 

Given that a notable number of aircrew members showed abnormal findings on SCH-1 or TBUT tests without reporting symptoms of dry eye, we conducted a multivariable logistic regression analysis to pinpoint independent risk factors for such positive findings in ophthalmological evaluations among participants with SPEED scores ranging from 0 to 6 (Table 3).

**Table 3:** Independent predictors of positive SCH-1 and TBUT results: multivariable logistic regression analysis TBUT: Tear break-up time; SCH-1: Schirmer’s-1

	Positive TBUT test			Positive SCH-1 test		
	Odds ratio	95% confidence interval	p-value	Odds ratio	95% confidence interval	p-value
Age, per one-year increase	1.05	1.01-1.08	0.01	1.03	0.99-1.07	0.06

In the multivariable model, age emerged as the sole independent variable predicting a positive TBUT test, with an OR of 1.05 and a 95% CI ranging from 1.01 to 1.08 (p = 0.01). This indicates that for each additional year of age, there is a 5% increase in the likelihood of a positive TBUT test. Additionally, age showed a borderline significant association with a positive result on the SCH-1 test, exhibiting an OR of 1.03 and a 95% CI between 0.99 and 1.07 (p = 0.06).

## Discussion

Our cross-sectional study provides valuable insights into the prevalence of subjective dry eye symptoms and their relationship with objective ophthalmological evaluations among civil aviation personnel. We analyzed a convenience sample of 189 workers during their routine fitness-for-flight visits and discovered three main findings. First, the majority of participants (n = 165; 87.3%) did not report any subjective dry eye symptoms, while 12.2% (n = 23) and 0.5% (n = 1) of the study subjects experienced moderate and severe symptoms, respectively. Second, 25.4% (n = 48) and 24.9% (n = 47) of the participants tested positive for DED on the SCH-1 test and TBUT, respectively. Interestingly, 18.8% (n = 31) and 17.6% (n = 29) of aircrew personnel who did not report subjective dry eye symptoms (SPEED scores between 0 and 6) showed abnormal results on the SCH-1 test and TBUT, respectively. Last, age was the only independent predictor of a positive TBUT, with a borderline significant association with a positive SCH-1 test.

In our study, the prevalence of moderate-to-severe subjective dry eye symptoms was 12.7%, notably lower than the 72.3% reported over two decades ago among 1246 Australian commercial pilots [13]. Nevertheless, the frequency of subjective dry eye symptoms in our study aligned with the reported prevalence of eye irritation (11%) among 1857 flying personnel of a Scandinavian airline [20]. Several reasons may account for such discrepancies, including different study periods, sample sizes, and participants' general characteristics. It is also possible that the improved controlled environment in the cockpit of newer aircraft - in terms of relative humidity conditions, localized airflow, and barometric pressure [21] - may currently exert less strain on the lacrimal functional unit of flying personnel compared to the findings of the study by McCarty and McCarty [13].

Nonetheless, it is remarkable that the prevalence rates of participants who tested positive for DED on the SCH-1 test and TBUT were higher (25.4% and 24.9%, respectively) compared to the rate of moderate-to-severe subjective dry eye symptoms according to the SPEED score (12.7%). Notably, a significant number of aircrew personnel without subjective dry eye symptoms in our study had abnormal results on the objective tests (18.8% for the SCH-1 test and 17.6% for TBUT). This discrepancy between subjective and objective assessments of ocular dryness in flying personnel is a critical aspect to consider as it suggests that individuals may report symptoms mainly according to their own sensitivity. Based on our findings, it is possible that civil aviation personnel tend to exhibit a “stoical” phenotype [22] when it comes to subjective symptoms, meaning they underestimate the dryness compared to the impairment shown on objective tests, rather than accurately or even sensitively reporting dryness. Intriguingly, a review by Alunno et al. [23] on dryness in primary Sjögren’s syndrome revealed that the “stoical” phenotype was more common in elderly patients with more severe disease. This led the authors to speculate that older individuals may develop better coping mechanisms for dryness over time. This possibility is in accordance with our data, which showed an independent association between age and positive results on the objective tests for airline personnel without subjective dry eye symptoms, specifically for TBUT and, to a lesser extent, for the SCH-1 test. These observations highlight the need to consider optimized strategies for screening and addressing this condition in aircrew personnel. In a recent secondary analysis of data from the Dry Eye Assessment and Management (DREAM) study, which focused on subjects with moderate to severe DED, Zhao et al. [24] found significant differences in objective signs of ocular dryness across different age groups. Hence, in light of the potential for underdiagnosis of DED if solely dependent on subjective symptoms, we propose the implementation of the SCH-1 test and TBUT as diagnostic tools for flying personnel aged 50 and above. By adopting this approach, we can greatly enhance the accuracy of identifying and treating DED through the use of artificial tears, prescription eye drops, tear stimulation techniques, and nutritional supplements.

Considering the potential detrimental impact of DED on the performance of aviation personnel, further research will be crucial to delve deeper into the pathophysiology of this condition and explore potential occupational countermeasures. Although artificial tears constitute the predominant treatment approach for ocular surface disorders [25], the unique environment of in-flight cabins poses a significant challenge for individuals requiring frequent eye lubrication. This is primarily due to the air at high altitudes being dehydrated as it passes through the aircraft's turbine. In this context, da Costa et al. [14] have previously demonstrated that certain commercially available bottles, equipped with a filtration membrane system, exhibit an inconsistent efflux of eye drops immediately after uncapping during flight. To ensure the ocular surface health of aviation personnel, the development of specifically formulated and packaged artificial tears for in-flight use will be of utmost importance.

There are several limitations to this study. First, the use of a cross-sectional design prevents the establishment of causality, limiting the research to identifying associations rather than making predictions or determining causation. Second, we did not conduct a comprehensive assessment of DED signs, such as conjunctival staining, corneal staining, meibomian gland dysfunction evaluation, and tear osmolarity. Third, the study relied on a convenience sample, potentially introducing selection bias. Finally, the single-center nature of the investigation may reduce the generalizability of the findings, necessitating confirmation in larger and independent samples.

## Conclusions

Our research offers significant findings on the occurrence of DED among civil aviation staff. The disparity between subjective symptoms and objective tests emphasizes the significance of incorporating objective measures for screening and diagnosing DED in civil flight personnel. If independently confirmed by future research, our findings could potentially lead to the routine implementation of surveillance protocols that incorporate objective DED indicators. Moreover, as age emerged as an independent predictor of positive results on objective tests, it is crucial to consider age-specific screening strategies.

## References

[1] Rouen PA, White ML (2018). Dry eye disease: prevalence, assessment, and management. Home Healthc Now.

[2] Tavares Fde P, Fernandes RS, Bernardes TF, Bonfioli AA, Soares EJ (2010). Dry eye disease. Semin Ophthalmol.

[3] Tsubota K, Pflugfelder SC, Liu Z (2020). Defining dry eye from a clinical perspective. Int J Mol Sci.

[4] Pflugfelder SC, de Paiva CS (2017). The pathophysiology of dry eye disease: what we know and future directions for research. Ophthalmology.

[5] Ngo W, Situ P, Keir N, Korb D, Blackie C, Simpson T (2013). Psychometric properties and validation of the Standard Patient Evaluation of Eye Dryness questionnaire. Cornea.

[6] Facchin A, Boccardo L (2022). Italian translation, validation, and repeatability of Standard Patient Evaluation of Eye Dryness (SPEED) Questionnaire. Cont Lens Anterior Eye.

[7] Schmidl D, Witkowska KJ, Kaya S (2015). The association between subjective and objective parameters for the assessment of dry-eye syndrome. Invest Ophthalmol Vis Sci.

[8] Hua R, Yao K, Hu Y, Chen L (2014). Discrepancy between subjectively reported symptoms and objectively measured clinical findings in dry eye: a population based analysis. BMJ Open.

[9] Saavedra Morales A, González Díaz CA, Villanueva López GC, Padilla Juárez O, Luna Torres AL, Sánchez Monroy V (2023). Prolonged computer use by office workers induces ocular symptoms associated with tear film alterations and overexpression of mucin 5 AC and catalase. J Occup Environ Med.

[10] Ay İE, Demirezen M, Şenol Y, Til A (2022). Ocular health among industrial workers: a prevalence study of foreign body injury, refractive error, dry eye, pterygium and pingueculae. Med Lav.

[11] Bazeer S, Jansonius N, Snieder H, Hammond C, Vehof J (2019). The relationship between occupation and dry eye. Ocul Surf.

[12] Uchiyama E, Aronowicz JD, Butovich IA, McCulley JP (2007). Increased evaporative rates in laboratory testing conditions simulating airplane cabin relative humidity: an important factor for dry eye syndrome. Eye Contact Lens.

[13] McCarty DJ, McCarty CA (2000). Survey of dry eye symptoms in Australian pilots. Clin Exp Ophthalmol.

[14] da Costa AX, Oliveira E Xavier LD, LaMonica LC, Dos Santos VR, Gomes JÁP (2021). Eye drop performance at high altitude: an "in-flight" problem. Eye (Lond).

[15] Li N, Deng XG, He MF (2012). Comparison of the Schirmer I test with and without topical anesthesia for diagnosing dry eye. Int J Ophthalmol.

[16] Yazdani M, Fiskådal J, Chen X, Utheim ØA, Ræder S, Vitelli V, Utheim TP (2021). Tear film break-up time and dry eye disease severity in a large Norwegian cohort. J Clin Med.

[17] Anantharaman D, Radhakrishnan A, Anantharaman V (2023). Subjective dry eye symptoms in pregnant women - a SPEED survey. J Pregnancy.

[18] Safarzadeh M, Heidari S, Azizzadeh P, Sheibani K, Nassiri N, Heidari L, Aghataheri S (2019). Comparative assessment of tear function tests, tear osmolarity, and conjunctival impression cytology between patients with pterygium and healthy. J Ophthalmic Vis Res.

[19] Roka N, Shrestha SP, Joshi ND (2013). Assessment of tear secretion and tear film instability in cases with pterygium and normal subjects. Nepal J Ophthalmol.

[20] Lindgren T, Norbäck D (2005). Health and perception of cabin air quality among Swedish commercial airline crew. Indoor Air.

[21] Bagshaw M, Illig P (2019). The aircraft cabin environment. Travel Medicine.

[22] Bezzina OM, Gallagher P, Mitchell S (2017). Subjective and objective measures of dryness symptoms in primary Sjögren's syndrome: capturing the discrepancy. Arthritis Care Res (Hoboken).

[23] Alunno A, Bartoloni E, Valentini V (2018). Discrepancy between subjective symptoms, objective measures and disease activity indexes: the lesson of primary Sjögren's syndrome. Clin Exp Rheumatol.

[24] Zhao M, Yu Y, Ying GS, Asbell PA, Bunya VY (2023). Age associations with dry eye clinical signs and symptoms in the Dry Eye Assessment and Management (DREAM) study. Ophthalmol Sci.

[25] Labetoulle M, Benitez-Del-Castillo JM, Barabino S, Herrero Vanrell R, Daull P, Garrigue JS, Rolando M (2022). Artificial tears: biological role of their ingredients in the management of dry eye disease. Int J Mol Sci.

